# Institutional guidelines and ongoing studies in management of liver tumours: the experience of the European Institute of Oncology

**DOI:** 10.3332/eCMS.2008.64

**Published:** 2008-05-02

**Authors:** R Biffi, F Orsi, MG Zampino, A Chiappa, N Fazio, F De Braud, G Bonomo, L Monfardini, PD Vigna, F Luca, L Bodei, M Bartolomei, G Catalano, MC Leonardi, M Ferrari, B Andreoni, A Goldhirsch, G Paganelli, R Orecchia

**Affiliations:** 1 Division of Abdomino-Pelvic Surgery, European Institute of Oncology, Via Ripamonti 435, 20141 Milan, Italy; 2 Unit of Interventional Radiology, European Institute of Oncology, Via Ripamonti 435, 20141 Milan, Italy; 3 Department of Medicine, European Institute of Oncology, Via Ripamonti 435, 20141 Milan, Italy; 4 Division of General and Laparoscopy Surgery, European Institute of Oncology, Via Ripamonti 435, 20141 Milan, Italy; 5 Division of Nuclear Medicine, European Institute of Oncology, Via Ripamonti 435, 20141 Milan, Italy; 6 Division of Radiation Therapy, European Institute of Oncology, Via Ripamonti 435, 20141 Milan, Italy; 7 Unit of Medical Physics, European Institute of Oncology, Via Ripamonti 435, 20141 Milan, Italy

## Abstract

**Background:**

An institutional task force on upper gastrointestinal tumours is active at the European Institute of Oncology (EIO). Members decided to collate the institutional guidelines on management of liver tumours (primary and metastatic) into a document. This article is aimed at presenting the current treatment guidelines as well as ongoing research protocols and trials in this field at the EIO.

**Methods:**

A steering committee convened to assign tasks to individual members. Contributions from experts in each treatment area were collated in a single document, in order to produce a draft for subsequent review from the aforementioned committee. Six drafts have been discussed and the final version approved.

**Results:**

Surgical, medical oncology, interventional radiology, nuclear medicine and radiation therapy approaches, their roles in management of liver tumours and ongoing research trials are presented and discussed in this article.

**Conclusions:**

At the EIO a multi-disciplinary integrated approach to liver tumours is standard and several ongoing research projects are currently active in this field.

## Surgery

### Introduction

Liver transplantation may overcome the problem of the low resectability rate in highly selected patients suffering from hepatocellular carcinoma (HCC), but it has very limited applicability, and non-resectional therapies with palliative (or, rarely, neo-adjuvant) intent currently provide the mainstay of treatment. Careful patient assessment is required to select those patients affected by colorectal metastases (CRMs), with good prospects of achieving substantial benefits by means of integrated treatments, including surgery, interventional radiology and chemotherapy (systemic and intra-arterial). A wide variety of treatment modalities are now available, each having its limitations, a number of which are reviewed here since they are routinely applied in our Institution. Such a wide range of treatment modalities requires a constant multi-disciplinary approach, where the management of each patient can be adapted to their particular needs. In the near future, the combination of these new technologies with advances in resectional surgery will offer significant improvements in the treatment of liver malignancies.

HCC is a global disease killing more than a million people each year, to whom surgery offers the only real chance of cure, even if the majority have an irresectable disease because of tumour stage or advanced cirrhosis [1]. Metastatic disease is the most common malignancy affecting the liver, and colorectal cancer is the primary source (CRMs) [2]. The management of hepatic malignancy has changed from a nihilistic approach to a more positive one; every patient should now be considered for curative resection, either at first presentation or after cytoreductive treatment to increase resectability rate. A better understanding of liver anatomy and physiology, the routine application of intra-operative US scan, the availability of new technological devices (ultrasonic dissector, bipolar electrothermal energy sealer, microwave coagulator, water jet dissector, ultrasonically activated shears and the harmonic scalpel), combined with general advances in intensive care, have recently improved the results of liver resection; mortality is now negligible in specialized centres, even in cirrhotic if well-compensated patients.

## Liver resection and combined non-surgical treatments

Hepatic resection is currently the only treatment able to offer a significant prognostic improvement in cirrhotic patients suffering from large HCCs, while liver transplant is still the best choice for very small lesions. Resection is also the best option for treatment of HCC developed in a normal liver [3,4].

In specialist centres, the surgical mortality rate of liver resection carried out in cirrhotic patients for HCC is under 3%; the five-year overall survival rate of these patients is more than 20%. In terms of crude survival rates, liver resection and transplant have the same results [5,6]. Nevertheless, disease-free survival is much better in patients undergoing liver transplant for a single, small HCC (less than 3 cm in diameter) [7]. Post-surgery recurrence of the disease is usually located in the liver, and occurs within two years of resection; sometimes it can appear as a metachronous lesion rather than a real recurrence of previous neoplasm [8]. Retrospective studies indicate a possible benefit from regular application of adjuvant drugs after curative resection [9]. In our experience, some patients could undergo radical surgery after a pre-operative neo-adjuvant treatment consisting of intra-arterial chemotherapy and embolization. Surgically controlled data [10] indicate that necrosis is obtainable with this approach in 40–100% of cases; in a pilot study carried out in 30 patients, an overall three-year survival rate of 60% was obtained in EIO.

A similar approach is currently proposed for surgical treatment of CRMs [11] initially unresectable in cases poorly located in the liver; the resectability rate is around 20% and five-year overall survival of these patients is 40%.

In highly selected patients, harbouring large solitary lesions, we perform an embolization of the portal branch affected by the CRM (usually the right one) to obtain, after 4–6 weeks, a compensatory hypertrophy of the remnant liver, thus avoiding the risk of post-operative liver failure. Another approach that we use regularly in selected cases is the so-called ‘repeat-hepatectomy’; based on the regenerative properties of the liver, it is possible to perform metastasectomies in different operations, even when liver deposits are found in most segments and a single operation is not initially feasible.

### 

#### Case study:

**Figure 3:**
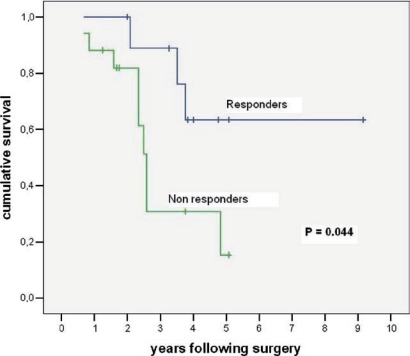
Chart showing relationship between responders and non-responders to neo-adjuvant chemotherapy and table comparing survival among patients undergoing resection of colorectal cancer liver metastases

Between December 1995 and May 2005, 88 patients affected by colorectal liver metastases underwent hepatic resection with curative intent. Twenty-seven of these patients (seven males, 20 females; mean age: 58 ± 8 years; range: 40–75 years) were treated with neo-adjuvant chemotherapy. A seven-year survival analysis was performed. Chemotherapy included mainly 5-fluorouracil, leucovorin, and either oxaliplatin or irinotecan for a median of eight courses. Results: 16 patients (59%) were affected by synchronous, and 11 (41%) by metachronous metastases. During pre-operative chemotherapy tumour regression was obtained in ten cases (37%), stable disease (SD) in another ten cases (37%), and progressive disease (PD) in the remaining 7 (26%). The five-year overall survival for CT responders was 64%, which statistically differed from non-responders at CT (15% at five years; p=0.044) (see Figure 3). Among all 27 resected patients, there was no perioperative mortality, and the major complication rate was 3%. Conclusions: the response to chemotherapy is likely to be a significant prognostic factor related to survival. Molecular markers associated with response and able to predict a clinical success for resection need to be defined.

A comparison between responders and non-responders to neoadjuvant chemotherapy is shown.

**Table:**
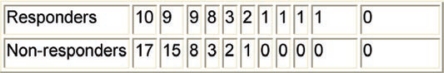

## New technologies and devices in liver resections

### Intra-operative ultrasonography

Ultrasound examination of the liver, at open operation or with laparoscopic access to the peritoneal cavity, is the most accurate determinant of the extent and location of intra-hepatic tumour deposits [12]. It is employed routinely in our institution, prior to resection or radiofrequency ablation, to define the extent of resection or ablation as well as to exclude small tumour deposits not defined by pre-operative studies.

Laparoscopic determination of incurability rather than determination at laparotomy results in less morbidity, lower costs and reduced hospitalization time [13]. Ultrasonography of the liver done at laparotomy may detect an additional 10–20% of tumour deposits [14], which were not visualized by conventional imaging methods, even if the introduction of a PET scan in the current clinical practice has recently reduced this rate. An additional advantage is the possibility of determining the location of a small tumour not palpable by the surgeon. Liver deposits are frequently missed in operations when simple inspection and palpation are used, especially when they are small in size and/or ill-located. Table 2 shows the results of a study carried out in our institution, demonstrating that up to 22% of liver metastases with a diameter less than 1 cm could not be located without intra-operative liver ultrasonography (IOLUS).

### Further new devices

Formal hepatic resection may be accompanied by considerable blood loss and the requirement for inflow occlusion, where ischaemia/reperfusion cycles and haemorrhage represent the major morbidity and mortality attributed to the surgery. Various techniques have been described to substantially reduce intra-operative blood loss during liver resection including the Cavitron ultrasonic aspirator (CUSA), microwave coagulation, water jet dissection, ultrasonically activated shears and the harmonic scalpel.

Each of these techniques may or may not be coupled with inflow occlusion or vascular isolation, potentially further impairing perioperative hepatic functional reserve. New techniques, which result in sealing during parenchymal dissection, will potentially result in less bile leakage and haematoma formation at the hepatic resection margin as well as less post-operative hepatic dysfunction. The use of ultrasonic dissection of liver tissue has become a standard option in our institution: Using this technique, damage to vessels and biliary ducts can be avoided, while hepatic parenchyma is separated and cleared through a cavitational effect which occurs at the tip of the vibrating rod of the device (25,000 cycles/sec), converting water to steam. The ultrasonic probe separates parenchymal cells because of their high water content by the cavitational effect without injuring structures having high content of fibrous tissue, like blood vessels and bile ducts.

Recently, a device which uses local bipolar electrothermal energy to seal small and medium-sized vessels (LigaSure^™^) has been safely used in haemorrhoidectomy [15], extended gastric cancer resection [16], limited pulmonary resections [17] and thyroidectomy [18]. Our group has carried out a study on this device in association with short periods of hepatic inflow occlusion, comparing this with the conventional finger-fracture technique of hepatic transection; assessing the safety and efficacy of the device, transection and total operative times and the effect on post-operative liver function after a range of formal and non-anatomical hepatic resections.

In this prospective study, we gained experience with the device in a consecutive series of 116 patients presenting with primary hepatocellular tumours (*n*=30), metastatic cancer (*n*=79) and benign lesions (*n*=7), all of whom underwent liver resections. A consecutive series of 63 hepatectomies were performed for various hepatic malignancies using the clamp crushing method with Pringle’s manoeuvre but without LigaSure diathermy (CC group). In this group, there were eight (13%) HCCs, 45 (71%) metastatic tumours, seven (11%) cholangiocarcinomas and three (5%) benign lesions. These cases were compared with 53 patients during this same time period including 12 HCCs (23%), 34 metastatic tumours (64%), three (6%) cholangiocarcinomas and four (7%) benign lesions who underwent hepatic resection using the clamp crushing method combined with Pringle’s manoeuvre and LigaSure sealing diathermy (CC-LS group). The LigaSure instrument uses bipolar electrothermal energy applied as a clamp to seal the target vessels. A feedback mechanism is incorporated into the device, which automatically stops the energy delivery when tissue sealing is complete, and the product works by obliterating the lumen and denaturing the collagen and elastin in vessel walls to create a bloodless permanent seal. Utilization of the LigaSure device in hepatectomy in combination with hepatic inflow occlusion proved to be safe with significantly less intra-operative blood loss and transfusion requirement without a substantial increase in transection time (see Table 3). This preliminary experience suggests an expanding role for bloodless hepatectomy and may assist in defining patient sub-groups where the application of the Pringle’s manoeuvre is unnecessary. The introduction of the LigaSure technique has specific advantages in laparoscopic surgery, where alternative energy sources to electrosurgery have been championed in association with an attempt to reduce instrument traffic. Our initial experience in formal and non-anatomical hepatic resection at open surgery would suggest that a prospective, randomized, controlled trial of its use with and without inflow occlusion is needed to define the clinical and operative benefits of this new methodology coupled with innovative heat sink strategies designed to lower temperatures at the instrument tip and to reduce thermal injury at the resection margins.

## Medical oncology

### Systemic chemotherapy of liver metastases from colorectal cancer

Colorectal cancer (CRC) is one of the most common human malignancies and remains a leading cause of cancer-related morbidity and mortality. Between 15% and 25% of patients have metastatic liver disease at diagnosis, and an additional 35–45% of patients will develop hepatic metastases during the course of their disease. Complete resection of hepatic metastases yields two- and five-year survival rates of 65% and 30%, respectively [19]. Seventy-five per cent of these patients will have a recurrence, 50% in the liver and 50% in extra-hepatic sites. Approximately 65–80% of all recurrences appear within two years. For patients who are not able to undergo liver resection, the five-year survival rate remains dismal and the function of chemotherapy is mostly palliative; the goal being to improve the quality and duration of life.

A modern era of colorectal cancer chemotherapy began in the mid-1990s, when the two novel agent oxaliplatin and irinotecan were found to have significant efficacy. These were the most substantive innovations in the field to occur since leucovorin was added as a biochemical modulator to fluorouracil (FU) in the early 1980s. The introduction of these drugs in clinical practice, for treatment of advanced disease, as infusional regimens like FOLFOX or FOLFIRI or, in combination with oral fluoropyrimidine, as XELOX or XELIRI, determined a gain in terms of objective response, from 23% to 50%, and overall survival, from 9–12 months to 16–19 months [20].

Neo-adjuvant chemotherapy has been explored in patients with unresectable liver metastases. Systemic chemotherapy containing irinotecan combined with fluorouracil/leucovorin enabled a significant portion (32%) of the patients with initially unresectable liver metastases to undergo liver resection. Also regimens containing oxaliplatin were able to induce successful resection in 41% of cases [21].

Progress in tumour biology research has improved the understanding of the important role of growth factors and their receptors in malignant transformation, tumour growth and metastasis, such that modulation of these factors may be an important anti-cancer strategy.

The epidermal growth factor receptor (EGFR) belongs to the erbB family of closely related cell membrane receptors: EGFR (HER-1), HER2, HER3 and HER4. In detail, EGFR is a widely expressed membrane spanning glycoprotein composed of three essential domains: extracellular, ligand-binding domain, hydrophobic transmembrane portion and cytoplasmic domain containing tyrosine kinase catalytic activity. The EGFR axis is activated by a variety of ligands including EGF and transforming growth-factor-alpha, that are crucial in the formation and propagation of many tumours, including colorectal cancer, through their effect on cell signalling pathways, cellular proliferation, control of apoptosis and angiogenesis.

Over-expression of EGFR is found in 65–70% of colorectal carcinomas but conclusive evidence as a prognostic indicator in this context is not available and deserves further evaluations [22].

Approaches to target the extracellular, ligand-binding domain include monoclonal antibodies such as cetuximab, that directly interfere with receptor signalling, while small molecule tyrosine kinase inhibitors (TKIs), such as gefitinib, are available with the capacity to interfere with catalytic activity and alter downstream signal propagation [23].

Cetuximab is a chimeric monoclonal antibody with a binding affinity for EGFR that is greater than naturally occurring ligands. This agent, combined with irinotecan, demonstrated a response rate of 23% in patients with EGFR-positive irinotecan refractory metastatic disease, versus 10% for cetuximab alone. Median time to progression was also significantly greater (8.6 versus 6.9 MOS) without survival benefit [24].

Gefitinib is an orally active EGFR tyrosine kinase inhibitor suitable for chronic once-daily dosing. No significant single agent activity was demonstrated but an interesting response rate was reported when combined to an oxaliplatin-containing regimen as second-line therapy.

We have recently conducted a phase II study: Gefitinib (Iressa®) in combination with FOLFOX4 regimen in EGF-positive advanced colorectal cancer not pre-treated for advanced disease.

The Italian, EIO-coordinated, multi-centric trial had four centres and 42 patients. After an encouraging 75% of objective response, the protocol was amended and 15 further cases were included to increase the statistical power. Final results confirmed the response rate but with a relatively short duration of response (median 7.1 MOS). No mature survival results are available yet. In our series, 14 patients became resectable after receiving this programme.

One main biological objective of this study was the serum extra-cellular binding domain of EGFR as a surrogate marker of tyrosine-kinase inhibition and as a predictor of tumour response. Higher serum EGFR was associated with the best objective response both at baseline and over time. This result was confirmed by a similar analysis, which considered the whole EGFR profile, instead of the basal value only (p=0.032). In our analyses, serum EGFR at baseline can be considered a significant predictor for the best objective response. This observation is in line with data reported on cell lung cancer [25]. Although the EGFR trend over time seems to confirm the basal difference, this result should be taken with caution, due to the small number of patients reporting EGFR values besides the basal one.

We have recently concluded enrolment for a randomized phase II first-line study with cetuximab (IgG1 monoclonal antibody, Erbitux) plus 5-FU/FA/oxaliplatin versus FOLFOX-4 in patients with EGFR-positive metastatic colorectal cancer (OPUS international trial). A total of 337 patients were treated in more than 70 centres in Europe (eight from EIO): Best response, percentage of resectable liver metastases determined by study treatment, progression-free and overall survival data are not yet available.

### Intra-arterial chemotherapy of liver metastases from colorectal cancer

Hepatic intra-arterial chemotherapy (HIAC) may be a better alternative to second- or third-line systemic treatments in patients whose disease is limited to the liver and who are refractory to systemic chemotherapy or at high risk of drug-induced toxicity [26].

The main concerns about HIAC are its high cost, the invasive nature of the procedure and the risk of extra-hepatic disease progression when using drugs with a high liver extraction rate such as fluoxuridine (FUDR). The delivery of drugs into the hepatic artery can be performed by means of different devices. Temporary catheters, usually inserted through the femoral artery, are removed at the end of the infusion. Arterial port-a-cath can be placed by surgeon or radiologist. In the first case a laparotomy is necessary. Radiologists can place the port in the chest inserting the catheter in the sub-clavian artery.

We used a percutaneous trans-sub-clavian temporary catheter placed by an interventional radiologist. Three drugs with a fair hepatic extraction rate and simultaneously reasonable systemic diffusion were chosen.

From September 1996 to December 1999, we treated 45 patients with 5-FU, CDDP and MMC within a clinical trial. A total of 33 patients had colon cancer and 12 had rectal cancer. All of the patients had been treated with 5-FU-based systemic chemotherapy, and 12 had also received other regimens. Twenty-four (53%) patients were refractory (nine to adjuvant treatment), and 21 (47%) were resistant. Twenty-four patients had received more than one previous systemic treatment. Twenty-five patients had more than three hepatic nodules. Twenty-five patients had synchronous and 20 metachronous metastases. Twenty-five patients had only liver metastases and 20 also had minimal extra-hepatic disease, mainly pulmonary. Four patients had primary tumour on site. Six patients had undergone liver metastasectomy. A total of 117 courses were administered: the median number per patient was three (range 1–5).

The study showed that HIAC, by means of a trans-sub-clavian percutaneous temporary catheter, is safe and active in pre-treated patients with metastatic CRC. Although it required careful monitoring because of the risk of tip displacement, laparotomy was avoided, and patient acceptance was satisfactory. Trans-sub-clavian, rather than femoral catheterization, allowed the patients to move freely during treatment. Systemic toxicity was very low.

Despite the fact that most patients were highly pre-treated and had extensive hepatic disease, 35% achieved a CR or PR (95% C.I. = 21.4–50.3%). Median overall survival was 11.7 months, and the one-year survival rate was 48%.

Furthermore, the cost of our therapy (US $14,216) was less than that reported for the pump infusion of FUDR through a surgical catheter (US $22,160) [33]. The oesophago-gastro-duodenoscopies performed in patients who complained of upper gastrointestinal symptoms after HIAC showed 20% of iatrogenic ulcers. The exact pathogenesis of this type of ulcer is unknown, but misperfusion has probably an important role. The risk of this complication therefore required strict abdominal x-ray assessment and made inpatient stay mandatory for each treatment. However, these ulcers did not cause any serious complications, always healed, and never contra-indicated the continuation of HAI.

Based on these encouraging results, we subsequently performed a study with the objective of investigating the role of HIAC containing FU plus folinic acid (FOLFai) combined with systemic treatment containing oxaliplatin (ivOX) alternating with FOLFOX-4 in patients with potentially resectable liver metastases from CRC. Nine patients were treated and three of them underwent radical surgery after response.

Although there is a compelling pharmacokinetic rationale underlying hepatic arterial infusion of fluoropyrimidine-based chemotherapy, this treatment is complicated, expensive and deserves a multi-disciplinary approach available in selected institutions.

### Metastases from breast carcinoma

Hepatic intra-arterial chemotherapy has been reported to produce higher response rate than systemic in patients with metastatic CRC. In metastatic breast cancer, the liver is involved in up to 60% of cases and often conditions the prognosis. Nevertheless, very little literature data about HIAC exist; systemic treatment remains the standard approach.

Based on our previous experience in primary and metastatic liver tumours, we performed a study of mitomycin, fluorouracil and cisplatin given through an arterial infusion. Eligible patients had progressive liver-dominant pre-treated disease. Twenty-eight female patients were treated, all having had received anthracyclines and all but four taxanes. Median age was 53 years. Ten had liver only and 18 liver-dominant disease. Time from liver metastases to intra-arterial chemotherapy was 33 months (range 7–110); time from diagnosis to liver metastases appearance was 34 months (range 6–168). Sixty per cent achieved a PR and 32% an SD. Latrogenic gastro-duodenal ulcer represented a severe but manageable complication. Liver-failure risk was high in extended liver involvement. Time to progression was around five months and overall survival 13 months. This is an inpatient treatment because of the percutaneous temporary catheter used. A new trial with a percutaneous radiological port-a-cath, earlier timing of therapy and different chemotherapeutic regimen has been planned.

### Hepatocellular carcinoma: systemic treatment

HCC is generally considered to be chemo-resistant. Responses to most single chemotherapeutic agents are uncommon. Although combination chemotherapy can produce a better response rate, toxicity is higher and evidence of improvement in survival remains elusive. Based on these reasons, new drugs are being used in the experimental setting in HCC. In particular, given that HCC is a highly vascular tumour, it is theoretically suitable for anti-angiogenetic therapy [27]. On this basis, we designed a clinical/biological study with thalidomide. So far only five phase I–II studies have been published, with less than 200 patients in total [28-32]. None of them considered biological objectives. In our trial, time to progression is the clinical objective and multi-modal anti-angiogenic activity evaluation the biological one. Perfusion CT scan, circulating endothelial cells, VEGF and bFGF blood levels and microvascular density, represent the different ways by which anti-angiogenic activity is measured. Pharmacokinetics is also studied. The trial is ongoing. So far 22 patients out of a planned 24 have been enrolled. Eligible patients must have an unresectable disease and must be not suitable for local–regional treatment such as radiofrequency (RF), trans-arterial chemo-embolization (TACE) and percutaneous ethanol injection (PEI). Neoplastic portal thrombosis does not represent an exclusion criterion, whereas patients with vascular thrombosis and any other cardiovascular diseases are excluded.

Some chemotherapeutic agents showed an anti-angiogenic activity when they are used at low doses in a metronomic fashion [33] (e.g. cyclofosfamide and methotrexate in breast cancer). On that basis, we are studying the ‘maintenance’ effect of capecitabine, at 2000 mg tot/day continuously, in advanced HCC patients. The objective of this study is to delay the progression of the disease, avoiding toxicity. So far 15 patients have been treated. One partial response, combined with a dramatic AFP decrease, was obtained in a HCV-related HCC patient with a total dose of 1000 mg/day of capecitabine two weeks out of three, because of baseline hyperbilirubinemia.

### Hepatocellular carcinoma: intra-arterial treatment

Primary liver malignancies are represented mainly by HCC (80%), and in a minority of cases by cholangiocarcinoma (10%) and gallbladder carcinoma (10%) [34].

Hepatic intra-arterial chemotherapy is potentially more active than systemic chemotherapy, because of the higher concentration of the drugs to the tumour bed, provided that the tumour blood supply is mostly arterial rather than venous [35].

Intra-arterial 5-fluorouracil (5-FU) or floxuridine (FUDR) produced 32% of response rate (RR) and a single agent such as cisplatin, which is relatively inactive in HCC patients when administered systemically, has reported response rate of 40–50% by intra-arterial infusion. Procedure invasiveness and clinical/haematological toxicity are the two main concerns of HIAC in HCC patients. The catheter is placed by means of a laparotomy in almost all studies, and the risk of toxicity is increased because of the underlying hepatopathy.

We studied a three-drug regimen administered IA consisting of mitomycin 2 mg/m^2^ (amended to 1 mg/m^2^) twice daily d1–3, cisplatin 10 mg/m^2^ twice daily d1–3, and 5-fluorouracil 1000 mg/m^2^/d as a continuous infusion d1–3, every six weeks for four courses. Catheters were inserted through the left sub-clavian artery and left on site over three days.

From 1997 to 2003, 44 patients were treated, 30 with HCC and 14 with biliary tract carcinoma. All patients had unresectable advanced disease, and 80% of HCC patients had cirrhosis. Eight partial responses and 21 SD diseases were obtained among 41 evaluable patients. Five out of the eight PRs were HCC. Median PR duration was six months, median SD duration was five months, time to progression (TTP) was four months, median overall survival (MOS) was 14 months, and one-year survival rate was 56%. Latrogenic gastro-duodenal ulcers occurred in about 10% of patients; grade 3 thrombocytopenia occurred in about 20% of cycles. In order to reduce the invasiveness of the procedure, we treated 11 patients with intra-arterial fluorouracil infused through a percutaneous trans-sub-clavian port-a-cath, combined with intravenous gemcitabine. This was an outpatient treatment and toxicity was acceptable. The activity results among the first 11 patients encouraged us to go on up to 30 patients, but the study is temporarily stopped because of kinking of the catheters.

## Interventional Radiology

### Introduction to the multi-modal approach for hepatic lesions

Hepatic lesions still represent a main clinical problem in oncology, both for primary and metastatic tumours. This is mainly due to the crucial role this organ represents in patient prognosis. In HCC, the simultaneous presence of cirrhosis and tumour lesions in the majority of patients limits the indication for an aggressive local approach, because of the high risk of post-treatment liver failure. Moreover, the high rate of new nodule development after any local treatment (>80% after four years) plays a key role in the decision making on treatment strategy.

Concerning liver metastases, in well selected patients, local treatments such as surgery for liver metastases (mainly from colorectal cancer) after systemic chemotherapy seems to induce better results than chemotherapy alone.

Minimally invasive local techniques are therefore being continuously developed in order to supplement the surgical resection.

### CT/PET in selection and follow-up after thermal ablation of liver metastases

Thermal ablation (RFA) is routinely employed in the treatment of focal liver lesions, mainly metastases from colorectal cancer. The main limitation of the percutaneous approach is still the inability to obtain data about treatment radicality compared to post-resection gross specimen analysis. In order to obtain much more fine detail about the ablated area, we recently introduced positron emission tomography (PET) in the early and late follow up of patients who have undergone RFA.

Our preliminary results on 20 patients have shown very important findings about the treated area including post-RFA residual pathological tissue and/or early local tumour relapse; no false-positive results have been documented, whereas CT detected some pathological findings many weeks later. Such early information plays a key role in the timely management of this subset of patients. We have an ongoing clinical trial on PET versus CT comparison in early and late follow-up after RFA of liver lesions.

### Percutaneous thermal ablation

HCC and colorectal liver metastases are the two most common malignant liver tumours. While surgical resection remains the gold standard of therapy, only a few patients are suitable candidates for curative surgical resection because of the presence of liver malignancy in unresectable locations, the number and anatomic distribution of tumour lesions, or the presence of extra-hepatic disease, poor liver function or medical co-morbidities. Percutaneous ablation using radiofrequency is a recently developed technique, proposed as a minimally invasive strategy for local treatment in patients with malignant liver tumours, whether primary or metastatic, who are not candidates for surgical resection. It induces temperature changes by using high-frequency alternating current applied via electrodes placed within the tissue to generate areas of coagulative necrosis and tissue desiccation. Radiofrequency ablation can be applied percutaneously, laparoscopically or at open surgery. From experiences reported in literature, despite the limitations of the data, reasonable safety of the procedure has been established, with mortality and morbidity rates in the largest series of 0.2% and 1.7%, respectively. The major complications described are peritoneal bleeding, hepatic abscesses, intestinal perforation, large biloma, acute cholecystitis. Tumour seeding is probably over-emphasized and can be avoided by a hot withdrawal of the electrode.

Candidate patients with HCC and cirrhosis have to be in Child’s class A or B; tumour has to be single or, if multiple, with no more than three nodules, without vein thrombosis or extra-hepatic spread [36]. The dimensions of the nodules should preferably be below 3–3.5 cm. By using combination therapy (hepatic artery occlusion and RFA; RFA and Pringle manoeuvre during surgery) larger tumours can be treated [37]. RFA is considered a safe bridge to liver transplantation in several recent papers [38].

In liver metastases, patients without a surgical prospect or refusing surgery were accepted for RF ablative treatment; indeed, RFA has lately been suggested for patients with resectable tumour [39]. The treated histotype was represented mainly by CRC, though other histotype metastases have been treated. Various investigators using percutaneous approach have adopted recruitment criteria for tumour dimensions and number that are the same as for HCC [40,41].

Pre-treatment work-up includes ultrasound (US) examination, unenhanced and contrast-enhanced CT or magnetic resonance (MR); serum tumour markers, namely alpha-fetoprotein (AFP) and carcinoembryonary antigen (CEA) dosage. Recently, FDG-PET examination has been introduced in staging liver metastases.

For HCC greater than 2 cm, two positive imaging techniques or one positive imaging technique and AF values higher than 400 ng/mL suffice for diagnosis and no biopsy is required. Usually a biopsy is performed in liver metastases, but when the history of the patient is well known biopsy could be left out of the pre-treatment work-up.

Before the procedure, blood-clotting tests are checked and patients with a platelet count below 40,000-50,000/mm^3^ and I.N.R. > 1.7 are refused.

Tumours close to the gall bladder or sub-capsular adjacent to hollow organs should be preferably treated by a laparoscopic or open approach; RFA of a lesion close to the hilum plate puts the patient at high risk of biliary injury.

Radiofrequency ablation is an effective procedure in focal lesion principally in small tumours no greater than 3–4 cm in diameter. Surgical resection was associated with a lower rate of recurrence and longer time to recurrence compared with RFA in treatment of HCC. However, surgery is usually performed in different patient groups, with RFA usually performed in patients who are unable to undergo surgical resection. Continued improvement in technology may also influence the success of RFA for treatment of liver tumours.

In our institute, we treated 89 patients, 64 with liver metastases (follow up 3–65 months, median 22) and 25 with HCC (follow up 2–63 months, median 23); RF was performed with ultrasound and/or CT guidance under conscious sedation and requires three days of hospitalization. In our experience, we had a very low percentage of complications, less than 1%, and no procedure-related mortality. To assess the results a contrast-enhanced CT is performed after one month: coagulation necrosis appears as a non-enhancing low density area in both arterial and portal phases; on MR T2 hypointense images and loss of enhancement on gadolinium-enhanced MR correspond to complete necrosis. FDG-PET allows for detection of pathological increase of glucose metabolism, as it happens in solid tumour. If a residual non-ablated tumour or tumour regrowth is detected a retreatment with RF can be performed.

### Hepatic intra-arterial chemotherapy

Since 1997 we have treated patients affected by liver neoplasms, primary and metastatic, with hepatic intra-arterial chemotherapy (HIAC) in our institution. In all these patients, we percutaneously implanted temporary hepatic artery infusion catheters under sonographic guidance using the sub-clavian artery. Main results have been presented in the part of this paper dealing with medical treatments.

### Super-selective hepatic chemo-embolization

Trans-arterial chemo-embolization (TACE) combines hepatic artery embolization with simultaneous infusion of a concentrated dose of chemotherapeutic drugs. Hepatic artery embolization refers to infusion of particles into tumour-feeding arteries without chemotherapeutic agents. Embolization by either technique renders the tumour ischaemic, depriving it of nutrients and oxygen. When chemotherapy is used, tumour drug concentrations are 1–2 orders of magnitude greater than are achieved by infusion alone, and the dwell time of the chemotherapy agent is markedly prolonged, with measurable drug levels present as long as one month later. Because most of the drug is retained in the liver, systemic toxicity is reduced. Interventional radiologists have derived therapeutic advantages from the dual blood supply to the liver and the propensity for neoplasm to derive their blood supply primarily from the arterial circulation: the celiac trunk and more peripheral hepatic vessels could be easily reached with trans-arterial catheterism for drug delivering.

Embolization and chemo-embolization lead to ischaemia of the tumour by blockade of the nutrient supply. An advantage of embolization is that the ischaemia induced by embolization helps to overcome drug resistance by causing metabolically active cell membrane pumps to fail, thereby increasing intra-cellular retention of the chemotherapeutic drugs. Recent research has demonstrated that ischaemia can increase angiogenesis in tumour cells, possibly spurring tumour growth. These molecular changes raise questions about whether chemo-embolization or hepatic arterial embolization is the better method to perform endovascular hepatic arterial therapy. To date, no study has demonstrated a difference in survival between the two techniques.

In our institution, the clinical role of TACE is different depending on specific disease; HCC and carcinoid liver metastases are the two main indications.

### Carcinoid liver metastases

Neuroendocrine tumours (NETs) represent a heterogeneous group of neoplasms originating from neuroendocrine cell compartments such as gastrointestinal and respiratory tracts. Their best combined indicators of prognosis and malignancy are evidence of invasive growth and presence of liver metastases [42,43].

Even though surgery is the only available curative treatment, successful management of disseminated NETs requires a multi-modal approach [44] that should be adapted to every single patient. Radiologists play a role not only in diagnosing but they can consider interventional options, such as TACE.

Seventy-one patients with NETs hepatic metastases were referred to our group for TACE between January 1996 and December 2006. During multi-disciplinary discussion, indication for TACE was given for every patient at a different time of the course of the disease, depending on the moment at which he could have the greatest advantage: the majority of patients (67/71, 95%) underwent first TACE and than radionuclide therapy in order to obtain a ‘de-bulking effect’ on liver lesions. They underwent 148 TACE as inpatients, ranging from one to a maximum six procedures. Treatment efficacy assessment was done after the last TACE or DOTATOC with abdominal CT or octreoscan: we observed 20 patients with responsive disease, 16 with stable disease, and 25 with progressive disease. Of those 25 patients, ten progressed for new hepatic lesions in spite of reduction or stableness of treated (TACE or DOTATOC) ones. In the last ten patients, the follow-up is still open.

In agreement with many current opinions, TACE will not have the function of exclusive treatment, but it could help to improve survival, and to control symptoms.

### Hepatocellular carcinoma

Chemo-embolization is accepted worldwide as an effective treatment for patients with unresectable HCC and adequate preservation of liver function; even in patients who are potential candidates for resection, chemo-embolization results in similar projected five-year survival rates compared with surgery (26% for chemo-embolization versus 42% for surgery; p = .556) for patients with a Cancer Liver Italian Program (CLIP: see below) score of 1 or higher.

In our institution from October 1994 to October 2006, 117 patients with histopathological diagnosis of HCC underwent 221 TACE; the numbers of procedures ranged from a minimum of one to a maximum of six and indication was given from multi-disciplinary group. A variable mixture of lipiodol ultra-fluid and farmorubucin was injected selectively (43% of TACE) or super-selectively (55%) in the pathological arterial vessel feeding HCC nodes; in only a few procedures (2%), treatment was given non-selectively in the right or left hepatic artery. All patients were treated as inpatients with a short hospital stay ranging from a minimum of three to a maximum of six days; no major treatment-related complications were observed, referring to the Society of Vascular and Interventional Radiology (SCVIR) classification.

Follow-up was made with a CT scan and clinical evaluation one, three and every six months after TACE. Any decision on whether to re-treat or follow-up was reached after case discussion. One- and two-year survival rates in our treated patients were 67% and 35%. Main reason of death was hepatic failure more commonly than HCC nodes progression. Based on the literature, we believe that our high survival rates probably depend on patient selection: during case discussions, we always took into account that general clinical parameters related to hepatic failure are often more relevant than nodes for patient prognosis. The Cancer Liver Italian Program (CLIP) scoring system is a medical HCC staging system that incorporates clinical and radiological parameters: when the score is higher than three in our discussion group, we suggest systemic therapy or supportive care rather than TACE or other invasive treatments.

### Yttrium-90 internal radiation therapy for hepatic malignancy

Surgical resection is the only potentially curative strategy in the treatment of patients with hepatic malignancy. Unfortunately, due to the advanced stage, underlying liver disease or medical co-morbidities, most patients are inoperable at this time of presentation. As a result, various loco-regional therapies have emerged for otherwise unresectable hepatic tumours. Intra-arterial administration of 90Y microspheres (SIR-Spheres) is a therapeutic option that allows preferential delivery of radiation into the tumour without significant liver toxicity [45]. SIR-Sphere (SIRTEX Medical, Australia) is a resin-based microsphere of 20–40-nm diameter, and a typical dose of 3–8 million per injection.

90Y is a pure beta-emitting radio-isotope, produced by the bombardment of 89Y with neutrons. 90Y has a high average energy (0.936 MeV), limited tissue penetration (mean 2.5 mm; maximum 11 mm) and short half-life (64 h), making it an ideal trans-arterial liver-directed agent. After incorporation into millions of resin microspheres, 90Y is selectively injected into the hepatic artery or one of its branches.

The administration of the microspheres is performed via a catheter placed in the hepatic artery. Since liver tumours are led by arterial rather than portal venous blood and microspheres are unable to transverse the tumour vessels, they remain trapped within the tumour and decay with the physical half-life of 90Y inducing irreversible damage in cancer cells.

Prior to the administration of radio-labelled microspheres, detailed imaging (CT, PET/CT) and therapy planning must be completed. All patients that are considered for 90Y internal radiation therapy must undergo a preliminary hepatic angiography in order to assess local vascular anatomy. Endovascular embolization of replaced hepatic arteries and/or non-hepatic collateral vessels (GDA, Right GA, etc) has to be performed in order to avoid extra-hepatic perfusion of radio-labelled particles. At the end of interventional vascular intervention, a 99mTc-macroaggregated albumin nuclear medicine scan is carried out. This work-up aims to delineate the hepatic arterial vasculature, parenchymal distribution, quantifying the degree of extra-hepatic and hepato-pulmonary shunt and calculating the appropriate dose of 90Y microspheres for the single patient.

This therapy is contra-indicated in patients with:

- previous external beam radiation therapy to the liver;
- ascites or clinical liver failure;
- markedly abnormal synthetic and excretory liver function tests;
- tumours amenable to surgical resection for cure;
- greater than 20% lung shunting (determined by the nuclear medicine break-through scan);
- pre-assessment angiogram and MAA nuclear scan demonstrates significant reflux of hepatic arterial blood to the stomach, pancreas or bowel;
- disseminated or main extra-hepatic disease;
- treatment with Capecitabine within the previous two months, or who will be treated with Capecitabine at any time following this treatment;
- portal vein thrombosis.

Studies in patients with metastases from colorectal cancer treated with SIRT demonstrated that 90% of patients had a post-treatment fall in serum levels of the tumour marker, carcinoembryonic antigen (CEA), and 82% of patients demonstrated at least some decrease in tumour volume as measured by serial computed tomography (CT) scans [46,47]. High response rates have also been reported using SIR-Spheres® to treat primary liver cancer. A trial of 71 patients with primary HCC treated with SIRT was recently reported.

Out of these, all 46 patients in whom elevated levels of the tumour marker alpha-fetoprotein were found at the pre-treatment, demonstrated a tumour marker reduction following the treatment and 89% had a greater than 50% decrease. In 27% of patients there was a 50% reduction in tumour volume.

#### EIO case study:

From October 2005 to December 2007, 16 patients with liver metastases (from colorectal cancer in six patients, breast cancer in three patients, uterine cancer in three patients, other cancers in five patients) have been treated with SIR-Spheres in our institute.

All patients but one (who had a previously implanted hepatic arterial port by means of percutaneous intervention) underwent diagnostic angiography through percutaneous sub-clavian artery access. Once vascular hepatic anatomy has been assessed, endovascular embolization of replaced hepatic arteries (e.g. right hepatic artery from SMA) or extra-hepatic arteries (e.g. GDA, right GA and/or hepatic/gastric trunk) are performed with endovascular metallic coils in order to obtain just one vessel feeding the whole liver parenchyma. At the end of vascular intervention, liver treatment is mimicked by injecting 99mTc-MAA through the vascular catheter located into the hepatic vessel which will be used for SIRT. The whole procedure is usually performed in outpatients.

**Figure 10:**
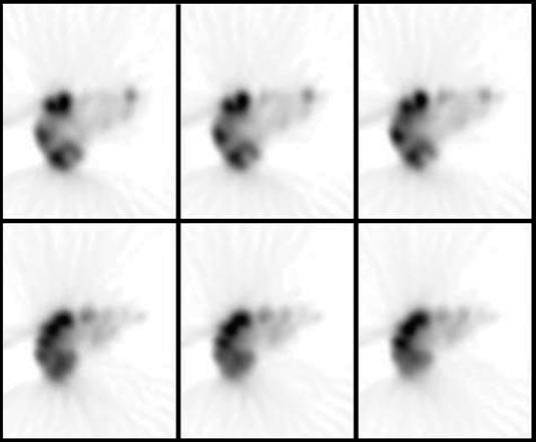
Preliminary SPECT scan after injection of Tc-macro-aggregated albumin in hepatic artery 99 m

For each patient, a dosimetric evaluation, based on 99mTc-MAA images (WB and SPECT, 30 min p.i.; see Figure 10) and CT scans, has been performed in order to estimate the activity to be safely injected. Image analysis determined the normal liver and lesions’ masses, the T/NT for all lesions and the possible shunt in lungs or GI tract. ROIs were concomitantly drawn on functional and anatomical images. To prevent toxicity, cautelative thresholds of 30–40 Gy in the normal liver, 20 Gy in the GI and in the lungs were established.

About two weeks later, once the appropriate activity was calculated, a single dose of 90Y microspheres was administered by a hepatic catheter, inserted through a percutaneous sub-clavian artery access, with the tip located in the same position used for MAA injection. Depending on the distribution of lesions, 90Y microspheres were delivered regionally (in three patients) or to the whole liver (the remaining patients).

In most cases, patients tolerated the procedure well and required only brief observation. Side effects were usually limited to nausea, vomiting, abdominal pain and transient elevations in liver enzymes. In two patients, a reversible gastro-duodenal ulceration was documented by endoscopy.

**Figure 11:**
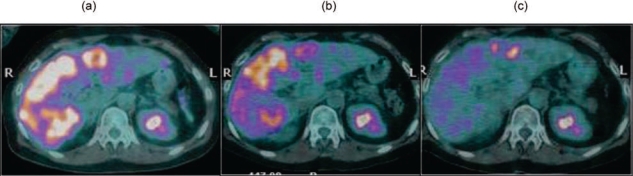
(a) basal CT-PET performed before SIR-Spheres therapy in patient with metastases from breast cancer shows huge masses into the hepatic right lobe; (b) CT-PET two months after treatment confirmed FDG uptake reduction; (c) CT-PET three months after treatment showed a very minimal residual tumour tissue still alive

Concerning the efficacy profile, revaluation CT and PET/CT (six, 12 and 18 weeks after treatment) documented objective response in 80% of patients (see Figure 11), with complete response in two of them.

Further prolonged follow-up will be mandatory in order to estimate the duration of the response and to answer three main issues: (1) What is the optimal dose and timing of 90Y microspheres? (2) What is the ideal mode of administration? (3) What factors predict successful outcome of 90Y radio-embolization?

### Pre-surgical portal vein embolisation for hepatic lobe hypertrophy

Portal vein embolization (PVE) before hepatectomy is aimed to induce a compensatory hypertrophy of the lobe to be preserved. PVE can induce hypertrophy of the future liver remnant volume resulting in a decrease of post-surgical risk of liver failure [48]. The procedure consists of ultrasound-guided percutaneous trans-hepatic puncture of one of the two intra-hepatic portal branches (contralateral to that to be embolized). After the direct portography has been performed, catheterization and embolization of the segmentary portal branches is obtained by using micro-particles (700–900 μm). The aim is to selectively reduce the portal flow, feeding the liver lobe to be resected, preserving portal circulation of the contralateral healthy lobe; lobe hypertrophy can be assessed with a CT scan, which is usually performed 40–50 days after PVE. In collaboration with the Division of General Surgery, we successfully treated six patients who underwent major liver resections, with preserved liver function after surgery.

## Nuclear Medicine

### Receptor radionuclide therapy of liver metastases from neuroendocrine tumours

Somatostatin receptors are over-expressed in many tumours, mainly of neuroendocrine origin, thus enabling primary and metastatic masses, mainly in the liver, to be localized by scintigraphy with the 111 In-labelled somatostatin analogue octreotide (OctreoScan®). The presence of somatostatin receptors is the basis for the subsequent treatment with ‘cold’ somatostatin analogues. Given the low response rate to this treatment, in the range of 5–7%, a new therapeutic approach using somatostatin analogues radio-labelled with suitable isotopes has been developed [49, 51].

Neuroendocrine tumours form a heterogeneous group including, as an example, gastro-entero-pancreatic and bronchial neuroendocrine tumours. They may present to the physician at different stages of disease, with or without associated hormonal syndromes. Functioning tumours are usually detected in earlier stages, due to hormone secretion rather than tumour bulk. Non-functioning tumours are usually diagnosed by the presence of a mass, along with distant metastases. Neuroendocrine tumours are frequently slow growing. In metastatic liver disease a multi-disciplinary approach, tailored to each patient, has been proposed to improve the therapeutic results. Receptor radionuclide therapy is one of the options, and is preferentially performed in minimal residual disease. Thus, our multi-disciplinary approach includes the symptomatic control of hypersecretory syndromes, with somatostatin analogues and/or interferon alpha-2b, and cytoreduction with surgery or TACE, in order to reduce the amount of tumour to be subsequently treated with radiopeptide therapy. New peptide receptor radionuclide therapy consists of the intravenous administration of a peptide, such as octreotide, labelled with a therapeutic radionuclide. The basis for receptor radionuclide therapy with radio-labelled octreotide in somatostatin receptor-rich tissues is the receptor-mediated endocytosis of the radiopeptide that is trapped inside the cytoplasm, thus allowing the irradiation of the cell.

Patients with evidence of disease at morphologic exams (CT, MRI or US), are selected for therapy only if diagnostic OctreoScan® images demonstrate an adequate uptake at the tumour site, at least identical to the physiological liver uptake, so that a low dose to normal organs and a high dose to the tumour can be predicted.

Labels currently used are the pure beta emitter 90Y ([90Y-DOTA0-Tyr3]-octreotide or 90Y-DOTATOC), and the beta-gamma emitter 177Lu ([177Lu-DOTA0-Tyr3]-octreotate or 177Lu-DOTATATE). The total amount of radioactivity is fractionated in several cycles up to a cumulative activity sufficient to irradiate the tumour but below the kidney or the bone marrow dose thresholds [52].

Yttrium-90 and Lutetium-177 are used as labels for their physical properties, which allow sufficient irradiation of the liver masses, due to a direct and a ‘cross-fire’ effect on surrounding receptor-negative cells (90Y: Emax 2.27 MeV; Rmax 11 mm; 177Lu: Emax 0.49 MeV; Rmax 2 mm). Our experience of receptor radionuclide therapy with 90Y-DOTATOC began in 1997, first with the dosimetric studies, then with two phase I studies (with and without renal protection with amino acids), followed by the evaluation of the response to therapy and the evaluation of kidney protection. In 2004, we started a phase I–II, open-label, non-controlled, two-step sequential study aimed at defining the efficacy and the acute and late toxicity of 177Lu-DOTATATE in patients affected by neuroendocrine tumours. The study is ongoing and to date we have enrolled the pre-fixed number of 51 patients.

### Safety aspects

With the pharmacokinetic and dosimetric studies, we established that both 90Y-DOTATOC and 177Lu-DOTATATE can be administered without causing serious toxicity [53]. The tumour receives high irradiation, while the bone marrow receives a low dose. Given its radiosensitivity at the activities used, the kidney represents the dose-limiting organ. Therefore, accurate renal protection schemes, based on amino acid co-administration, are used, in order to lower the dose to the kidney and to reduce the risk of permanent renal toxicity. No endocrine dysfunction of pituitary axes (thyroid, adrenals, gonads) nor diabetes mellitus was observed after radiopeptide therapy.

### Efficacy

Dosimetric studies indicate that 90Y-DOTATOC and 177Lu-DOTATATE are suitable for efficient receptor radiotherapy. High absorbed doses to tumour lesions (>80–100 Gy) may result in a high percentage of cure [54]. In our clinical trial of receptor radionuclide therapy with 177Lu-DOTATATE, amongst the 24 patients who performed the evaluation exams, complete response was observed in one patient (4%), partial response in five (21%), minor response in five (21%) and stabilization in 13 (54%). Responses lasted 4–25 months (median 18), while the follow-up lasted 6–38 months (median 21). From the evaluation of the objective response of 141 patients with somatostatin-receptor-positive tumours of various origins, treated with cumulative activities of 7.4–26.4 GBq of 90Y-DOTATOC, we observed a 26% objective response (PR+CR), with duration of responses ranging between two and 59 months (median 18). Patients who responded were affected mainly (69.7% of cases) by gastro-entero-pancreatic neuroendocrine tumours.

We selected and treated with 90Y-DOTATOC a group of 114 patients affected by pure neuroendocrine tumours mainly of gastro-entero-pancreatic origin, the large majority of which (94%) with progressing liver metastases. Liver metastases developed from bronchial (*n*=14), gastric (*n*=3), small intestine (*n*=34), rectum (*n*=3), functioning (*n*=16) and non-functioning (*n*=29) pancreatic, and unknown origin (*n*=14) endocrine carcinomas. Patients received cumulative activities of 90Y-DOTATOC ranging from 7.4 to 38.2 GBq, in two to 12 cycles. In a 5–81-month follow up, objective responses (CR+PR) were observed in 42 patients (38%), while 58 patients (50%) stabilized. Responses ranged from three to 76 months (median 20).

## Radiotherapy

Historically, liver radiation therapy has been limited by the low tolerance of parenchyma to radiation. Doses greater than 30 Gy, delivered over the whole organ are highly associated with the development of a clinical syndrome radiation-induced liver disease (RILD), which is a progressive, vascular-based entity, potentially degenerating to cirrhosis and liver failure [55]. Thus, the irradiation of the whole liver is mainly used as a palliative treatment, in case of massive liver involvement; radiotherapy doses of 20–30 Gy are used to obtain an effective relief from pain, although patient’s survival does not increase consistently (median: 3–9 months). Challenging integrating treatments, using unconventional radiotherapy fractionation schedules, associating radiations with radiosensitizer drugs have produced conflicting results, without significant improvements of clinical outcome. Better results seem to come from the integration of radiotherapy with intra-hepatic arterial infusion of 5-fluorouracil and 5-fluorodeoxiuridine; higher response rates, up to 85%, have been obtained compared to the medical treatment alone [56].

Radiobiologically, the liver is characterized by a physiological parallel architecture; meaning that a close relationship exists between radiation absorbed dose and irradiated volume in terms of risk of functional damage. Thus, it is possible to deliver high radiation doses to a small portion of liver parenchyma without excessive risk of hepatic failure.

The development of highly selective radiotherapy techniques, like three-dimensional conformal therapy, recently allowed the irradiation of smaller portion of liver parenchyma with higher doses, with similar risk of developing RILD, but higher chances of obtaining a ‘curative’ effect. It is possible to deliver, in a liver irradiation course, doses higher than 70 Gy, allowing treatment enhancement and dose-escalation protocols. Published series have reported local control rates of 68% and median survival periods ranging from 16 to 20 months.

Furthermore, interest lies in a specific partial liver irradiation modality: stereotactic hypofractionated radiotherapy [57]. It may represent an effective irradiation approach, delivering therapeutic doses in a single or a few treatment fractions, with practical advantages of patient compliance, better integration with other treatment modalities, and comparable outcome in terms of toxicity and local control. Reported data seem to confirm the potential value of this modality with three-year tumour control rates of almost 70%, and no major acute and late side effects.

For this irradiation modality, it is mandatory to guarantee an accurate target definition and adequate treatment reproducibility. Thus, specific efforts are required to guarantee improved patient setup and target localization, as well as to minimize, through specific systems of breathing control, tumour respiration-related motion.

At the EIO, since May 2003, the Radiotherapy Division has been exploring this kind of treatment for primitive and metastatic liver cancer localizations, with particular efforts in patient selection, and integration with other loco-regional treatments. Specifically, treatment procedures: dose and fractionation schedules have been investigated, as well as the implementation of breathing control modalities. Forty-eight stereotactic liver treatments have been performed in 40 patients (male/females: 16/24), both for primary HCC (five) and for metastatic liver localizations (43). In 19 cases, treatments have been performed with a declared palliative intent; otherwise, in 29 cases both the limited number (no more than three synchronous lesions at the time of irradiation), and the small size of lesions (less than 8 cm, measured in long axis), as well the absence (or control) of extra-hepatic disease, configured a ‘curative’ purpose (see Table 7).

Irradiation doses ranged from 15 Gy in a single fraction (two cases) to 36 Gy in three fractions; the majority of patients being treated with total doses exceeding 24 Gy. Compliance to treatment has been satisfactory and acute toxicity was confirmed to two cases of gastric bleeding, due to the close position of the treated lesion to the stomach or the small bowel. As far as ‘curative’ treatments are concerned (29 treatments), 22 cases are actually evaluable for response to treatment, having a minimal follow-up of three months; for them, the first site of eventual disease progression (PD) has been evaluated in order to define the possible efficacy of stereotactic irradiation. In ten cases, no PD occurred at all (45%); in the remaining five cases (23%) experienced a PD at the site of irradiation (in two cases as a unique progressive disease), while seven cases (32%) evidenced a PD in another part of liver parenchyma (four cases) or as a systemic diffuse disease (three cases) but not at the site of treatment (see Table 8). Interestingly, examining the sub-group of treatments with the highest doses (seven cases, total dose: 36 Gy), local control was consistently better, with five cases (71%) without PD at all and two cases (29%) experiencing a PD in other part of liver parenchyma. This observation strongly supports a dose–response effect, underlining the importance of accurate patient selection and treatment implementation. These preliminary data confirm the feasibility of the modality, although extensive follow-up data are still warranted to explore the potential value of stereotactic irradiation on overall clinical outcome.

It is important to remember that stereotactic radiotherapy, as well as other high-dose partial liver irradiations, should not be considered antithetic to other loco-regional approaches, but as a complementary treatment. Thus, clinical research should be dedicated to the optimization of the technical aspects, and at the same time to the evaluation of the specific selection criteria for the different therapy options and for their correct integration.

## Discussion and conclusions

### Colorectal liver metastases

The criteria for resectability of colorectal liver metastases have been significantly revised and expanded over the past decade, and factors such as the tumour number, tumour size and presence of extra-hepatic disease should no longer be used to exclude patients from consideration for surgical resection, especially when a combined treatment can be proposed. Combining hepatic resection with radiofrequency ablation can expand the number of patients who may be submitted to liver surgery, particularly as larger lesions that are less effectively treated with ablation can be resected and small lesions can be ablated. Radiofrequency ablation has applicability in patients who do not meet the criteria for resectability but are candidates for liver-directed therapy based upon the presence of liver-only disease. In those patients with extensive disease and limited hepatic reserve, portal vein embolization or a two-stage hepatectomy approach can sometimes permit surgical resection. Similarly, the use of pre-operative chemotherapy can be associated with conversion of some patients from unresectable to resectable, but perhaps more importantly pre-operative chemotherapy can help assess the underlying biology of the patient’s disease. Patients with extensive extra-hepatic disease, or those who progress on systemic chemotherapy, should probably not be considered for hepatic resection. Over the past decade, chemotherapeutic and biologic agents have been found having significantly greater activity against colorectal cancer. These newer agents have led to higher response rates, exceeding 50% with combination therapies that include oxaliplatin and irinotecan. This superior efficacy of chemotherapy agents has allowed the treatment of a subset of previously unresectable patients and has ‘converted’ them so that they can undergo liver surgery following tumour downsizing. About 15–20% of patients with initially unresectable disease have significant tumour downsizing to the point that the metastatic disease can ultimately be considered resectable. As such, patients with extensive disease who respond to chemotherapy should be re-assessed by a hepatic surgeon. By reconsidering the initial unresectability of patients, hepatic resection and long-term survival may be achieved in a sub-group of patients who otherwise would have a poor outcome. Ultimately, a therapeutic approach that includes all aspects of multi-disciplinary and multi-modality care is required to select and treat this complex group of patients.

### Hepatocellular carcinoma

Patients with HCV-related HCC have generally a better liver function than alcoholic patients, and are less prone to develop liver failure after aggressive procedures. This fact might explain the better results of arterial chemo-embolization in patients with viral disease compared with patients with alcoholic cirrhosis. Conversely, it has also been suggested that hepatic recurrences after surgery or ablative therapy are more frequent in patients with HCV-related HCC, possibly due to a higher incidence of new tumours. HCC screening is now widely performed in industrialized countries, leading to a better knowledge of liver carcinogenesis and an increasing rate of small HCC at diagnosis. Important advances have occurred concerning curative treatment of small tumours; even if liver transplantation, which is the best curative option for the long term as it is able to remove the tumour and the underlying cirrhosis (preventing therefore the occurrence of new tumours), can be performed in only limited number of well-selected patients. Other curative options, resection and percutaneous ablation mostly by radiofrequency, are able to cure the tumour mostly when small and well circumscribed. Due to a lower mortality and morbidity and a less deleterious influence on liver function, radiofrequency is to be performed in an increasingly larger number of patients with cirrhosis as new techniques now allow treatment of tumours more than 3 cm in diameter. Nevertheless, tumour recurrence rate is high (10–20% per year) in cases of resection or radiofrequency due to either intra-hepatic metastasis or occurrence of a new HCC, a case that is particularly frequent in patients with HCV infection. This fact justifies post-therapeutic surveillance of patients and the search for preventive treatments.

### Neuroendocrine tumours liver metastases

In metastatic liver disease, a multi-disciplinary approach, tailored to each patient, has shown an improvement in the therapeutic results. Receptor radionuclide therapy is preferentially performed in minimal residual disease. Thus, our multi-disciplinary approach includes the symptomatic control of hypersecretory syndromes, with somatostatin analogues and/or interferon alpha-2b, and cytoreduction with surgery or chemoembolization, in order to reduce the amount of tumour to be subsequently treated with radiopeptide therapy using 177Lu-DOTATATE.

### Radiation therapy

The development of highly selective radiotherapy techniques, like three-dimensional conformal therapy, recently allowed the irradiation of a smaller portion of liver parenchyma with higher doses, without excessive risk of hepatic failure. Stereotactic hypofractionated radiotherapy may represent an effective irradiation approach, delivering therapeutic doses in a single or a few treatment fractions, with practical advantages of patient’s compliance, better integration with other treatment modalities, and comparable outcome in terms of toxicity and local control, both for hepatocellular carcinoma and colorectal liver metastases. Preliminary data confirm the feasibility of the modality, although extensive follow-up data are still warranted to explore the potential value of stereotactic irradiation on overall clinical outcome.

In conclusion, an institutional task force on upper gastrointestinal tumours is active at the EIO. Members decided to collate the institutional guidelines on management of liver tumours (primary and metastatic) into a document. Contributions from experts in each treatment area were collected in a single document, in order to produce a draft for subsequent review from the aforementioned committee. Six drafts have been discussed and the final version approved, representing the content of this article. Surgical, medical oncology, interventional radiology, nuclear medicine and radiation therapy approaches, their roles in management of liver tumours and ongoing research trials have been presented here and discussed, focusing on the value of the multi-disciplinary approach to liver neoplasms.

## Figures and Tables

**Figure 1:**
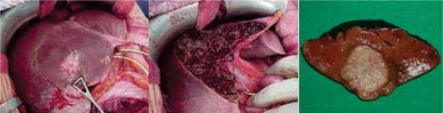
(a) wide excision of a superficial colorectal metastasis of the quadrate lobe of the liver; (b) resectional margin appearance; (c) gross pathology finding

**Figure 2:**
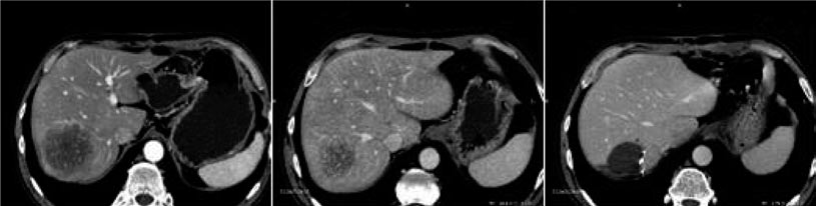
(a) CT before combined systemic and intra-arterial chemotherapy—large lesion the right hepatic lobe, more than 7 cm in diameter; (b) CT after two cycles of combined chemotherapy—the lesion is smaller, with a diameter of less than 4.5 cm; (c) CT after right hepatic lobe resection: post-surgical small bile collection (arrow) and the evidence of left hepatic lobe hypertrophy

**Figure 4:**
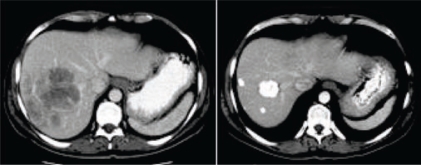
Patient with a partial response to i.a./i.v. chemotherapy. (a) pre-treatment CT shows a huge lesion into the right lobe; (b) post-treatment CT confirms lesion reduction, detecting some calcifications within the treated lesions

**Figure 5:**
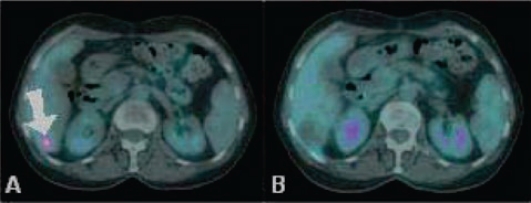
(a) CT/PET before percutaneous thermal ablation of hepatic metastasis from colorectal cancer—FDG uptake by the lesion (arrow); (b) CT/PET performed six months later percutaneous treatment, clearly shows the absence of activity by the lesion (no FDG uptake)

**Figure 6:**
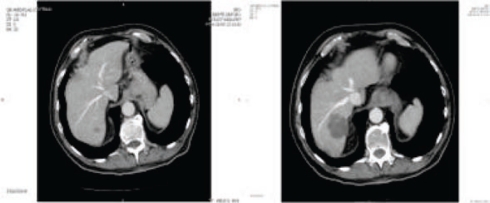
Liver metastases from CRC in segment 7: (a) CT before treatment; (b) post-treatment CT shows a low-density area (= necrosis) where thermal ablation has been performed

**Figure 7:**
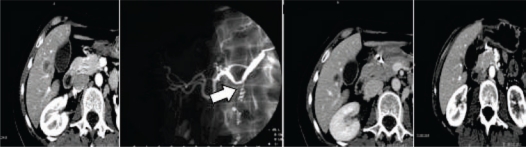
(a) CT scan showing hepatic lesion within VI segment, before treatment; (b) Hepatic angiography after coil-embolization, shows complete occlusion of GDA (arrow); (c) CT scan after first cycle of HIAC: shrinkage of the lesion; (d) CT scan after three cycles of HIAC: further shrinkage of the target lesion. Patient affected by breast cancer metastases.

**Figure 8:**
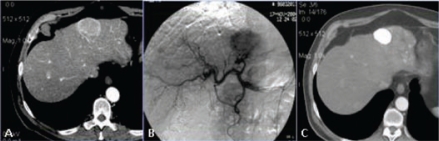
(a) pre-TACE CT shows a mild hypervascularity around the NET liver metastasis to be treated; (b) hepatic artery angiogram confirms the lesion, fed by the left hepatic artery; (c) post-TACE CT shows the good result after treatment, with a homogeneous Lipiodol uptake and an initial shrinkage.

**Figure 9:**
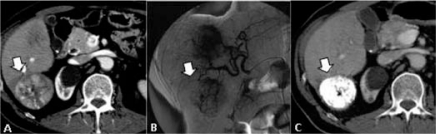
(a) pre-TACE CT shows a huge hypervascular nodule of HCC located into the segment VI; (b) hepatic angiogram detects many arteries feeding the lesion to be treated; (c) post-TACE CT shows the intense Lipiodol uptake into the treated lesion, which is a little bit smaller after one month.

**Figure 12:**
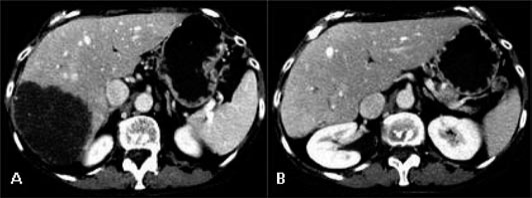
(a) pre-surgical CT shows a huge liver lesion; (b) CT after resection performed after embolization of right portal branch, confirms liver left lobe hypertrophy

**Figure 13:**
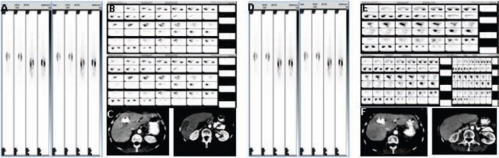
Best objective response in a patient affected by liver metastases from non-functioning endocrine pancreatic carcinoma, treated with 90Y-DOTATOC. At the enrolment the patient had progressed elsewhere after trans-arterial chemo-embolisation, performed six months before (a, b and c: basal whole-body scan, anterior and posterior view, SPECT sections and CT sections, respectively; d, e and f: whole-body scan, anterior and posterior view SPECT sections and CT sections after therapy).

**Figure 14:**
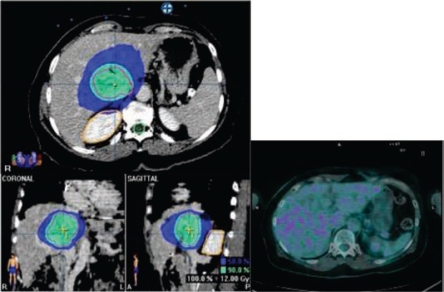
(a) stereotactic irradiation of CRC metastatis (total dose 36 Gy); (b) two-month follow-up PET/CT: no evidence of disease

**Figure 15:**
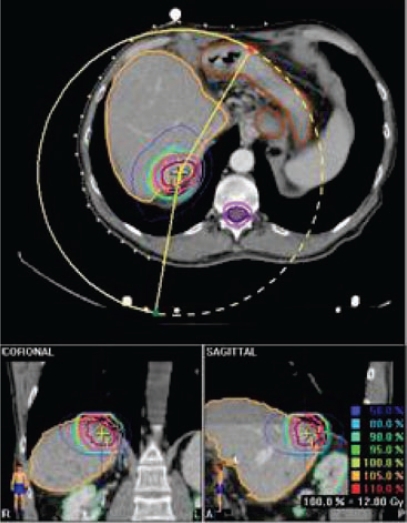
Selective stereotactic irradiation of hepatic metastatis from CRC

**Table 1:**
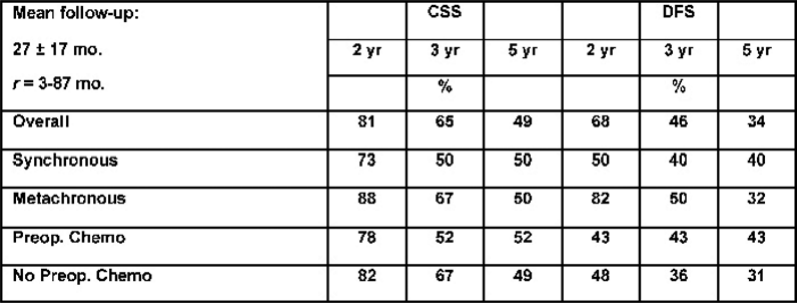
EIO experience in liver resections for colorectal cancer metastases: survival rates—88 pts

**Table 2:**
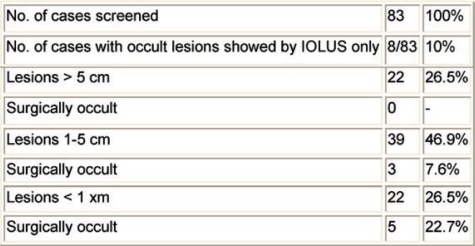
The value of intra-operative liver ultrasonography (IOLUS) in diagnosing liver metastases from colorectal cancer

**Table 3:**
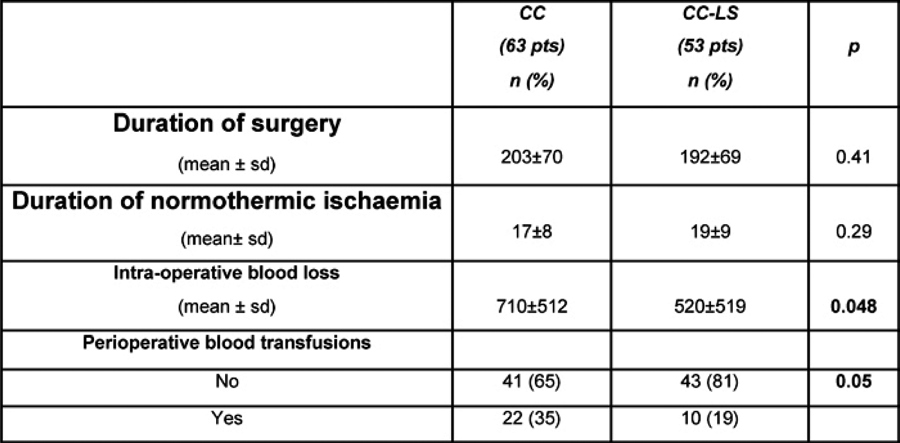
Surgical outcomes comparing traditional clamp crushing resection technique (CC) and the LigaSure-assisted one (CC-LS)

**Table 4:**
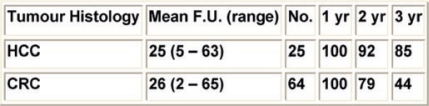
Data about our own experience about percutaneous thermal ablation of hepatic lesions: survival rates—89 pts

**Table 5:**
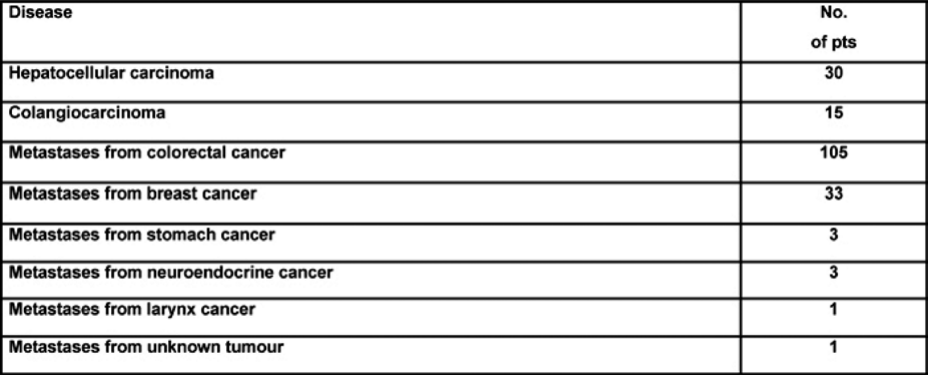
Number of pts treated with HIAC, and histology of the liver lesions

**Table 6:**
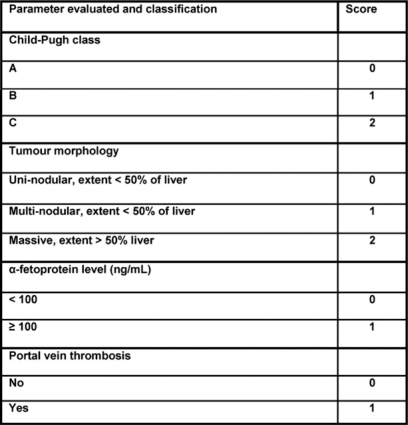
CLIP scoring system

**Table 7:**
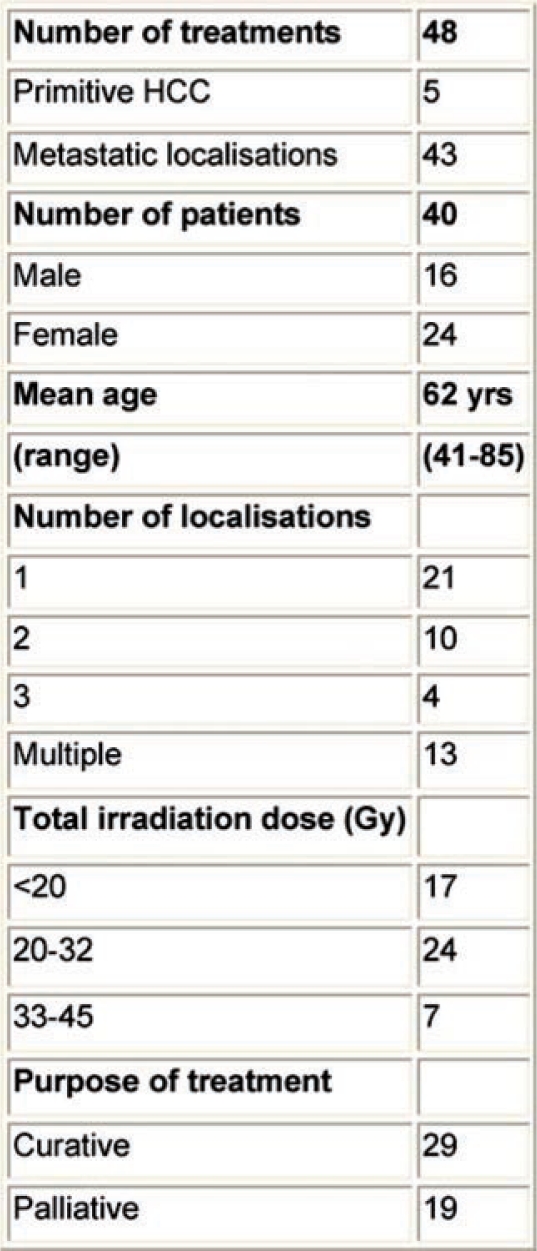
Characteristics of population—hypofractionated stereotactic radiotherapy in primitive and metastatic liver cancer localizations

**Table 8:**
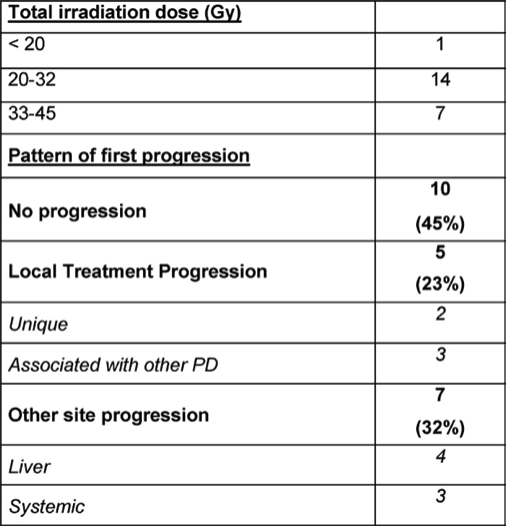
Treatment dose and patterns of failure for curative irradiation—curative stereotactic irradiation with minimal follow-up of three months (22 treatments)
